# Health-Related Quality of Life of Spinal Cord Injury Patients Visiting the Outpatient Clinic of a Tertiary Care Rehabilitation Center in Saudi Arabia: Results from a Cross-Sectional Study

**DOI:** 10.3390/healthcare14070881

**Published:** 2026-03-30

**Authors:** Anas J. Alsaleh, Shabana Tharkar, Khalid M. Almutairi

**Affiliations:** 1 Physical Medicine and Rehabilitation Unit, Sultan bin Abdulaziz Humanitarian City, Riyadh 13248, Saudi Arabia; dr.anas.rj@gmail.com; 2 Community Health Science Department, College of Applied Medical Science, King Saud University, Riyadh 12372, Saudi Arabia; stharkar@ksu.edu.sa

**Keywords:** spinal cord injury, quality of life, Saudi Arabia

## Abstract

Background: Spinal cord injury (SCI) patients face many difficulties in life, affecting their quality of life. This study evaluated the health-related quality of life (HRQoL) of patients with spinal cord injury in Saudi Arabia and additionally identified the key determinants associated with lower scores. Methods: This cross-sectional study recruited 50 SCI patients from an outpatient clinic of a tertiary care rehabilitation center during the last quarter of 2024. The WHOQOL-BREF questionnaire was used to assess the HRQoL across overall, physical, psychological, social, and environmental domains. The scale of scores was transformed to 0–100 using the formula. Descriptive statistics were used to present categorical variables in terms of frequencies and percentages, and continuous data as means and standard deviations. Inferential statistics were used to determine the relationship between the various dependent and independent variables. Results: Of the total sample of 50 SCI patients, 84% were caused by motor vehicle accidents, and 74% were at the paraplegia level. The mean age was 35 ± 10.46 years, the average time since the injury was 6.3 ± 6.0 years, and the mean number of admissions of the study patients was 3.2 ± 1.6. The overall HRQoL score was 75.5 ± 24.5, and the general health score was 66.5 ± 31.0. Among all the WHOQOL-BREF domains, the psychological domain achieved the highest score (71.7 ± 17.5), while the physical health domain scored the lowest (55.9 ± 18.8). Presence of neuropathic pain was significantly associated with lower scores across overall quality of life (*p* = 0.033), physical health (*p* = 0.022), and psychological health (*p* = 0.044). A notable correlation was identified between poor environmental health and the presence of spasticity (*p* = 0.042). Depression was significant (*p* = 0.047) in patients with low physical health scores. Conclusion: Neuropathic pain, spasticity, and depressive symptoms were the strongest determinants of lower HRQoL, indicating the importance of targeted multidisciplinary management.

## 1. Introduction

Spinal cord injuries (SCIs) represent severe traumas that can lead to considerable physical, psychological, and socio-economic consequences. These injuries result from damage to the spinal cord, which interrupts nerve communication between the brain and the body Consequently, this results in a range of medical issues and complications, such as paralysis, sensory loss, bowel and bladder dysfunction, pain, pressure ulcers, and spasticity, all of which can adversely affect the quality of life for individuals suffering from spinal cord injuries [1,2]. The severity of impact is directly proportional to the severity and the level of the injury requiring rehabilitation and long-term management. Rehabilitation for spinal cord injury patients generally adopts a multi-disciplinary strategy, encompassing physical therapy, occupational therapy, and psychological assistance to enhance functional recovery, minimize secondary complications, and enable individuals to achieve the best possible quality of life [3,4,5].

The global burden of SCI is estimated to be approximately 15 million cases, of which nine million are males [6]. Primarily, the chief cause of spinal cord injuries is road traffic accidents, followed by falls, sports-related injuries, and gunshot wounds [7]. The annual incidence of spinal cord injuries in the Middle East and North Africa (MENA) region varies by country. The estimated SCI prevalence ranges from 7.8 per million in Kuwait to 72.4 per million in Iran, with a pooled prevalence of approximately 23.24 per million people [7]. Saudi Arabia has one of the highest rates of spinal cord injuries globally, with approximately 62 cases per million, predominantly due to traffic accidents [8].

The health-related quality of life (HRQoL) in SCI patients is a complex and multifaceted concept that encompasses both objective elements (such as disability, pain, and fatigue) and a personal assessment of how these elements impact one’s overall happiness [9]. According to the World Health Organization (WHO), quality of life is defined as: “*individuals’ perception of their position in life within the cultural and value frameworks they inhabit, and in relation to their goals, expectations, standards, and concerns*” [10]. Given its intricate effects on the human body and its links to various physical and psychological issues, it has been observed that patients with spinal cord injuries experience a diminished HRQoL [11].

Studies examining the health-related quality of life in people with SCI have shown low HRQoL compared to the general population. The main factors contributing to decreased HRQoL were identified as pain and dependence on assistance for mobility [12], followed by issues like health problems, employment status, work-related stress, and educational level [13,14]. Although extensive research has been conducted worldwide on spinal cord injuries, there remains a significant lack of studies focused specifically on Saudi Arabian patients, especially concerning their health-related quality of life after injury. Al-Jadid’s research on the quality of life for individuals with SCI found that financial situation, employment, access to necessary equipment, and social isolation are directly linked to their HRQoL [15]. Al-Oweisi’s study of anxiety and depression levels within the SCI community in Saudi Arabia revealed that women with SCI experience higher levels of anxiety and depression, along with other factors like pain and educational background [16].

There is a paucity of data related to HRQoL in SCI patients in Saudi Arabia. Understanding the health-related quality of life is essential for improving rehabilitation outcomes and overall patient management. This emphasizes the need for focused research to evaluate the HRQoL among SCI patients. Therefore, the objective of this study was to assess the health-related quality of life in SCI patients in Saudi Arabia and to identify the factors associated with lower HRQoL. This study adds important information on HRQoL in SCI patients and its key determinants.

## 2. Methods

### 2.1. Study Design and Setting

The study was carefully designed and conducted following STROBE guidelines. This cross-sectional study was conducted at the outpatient clinic of a large tertiary care medical and rehabilitation center during September and October 2024. The target population included individuals with traumatic spinal cord injuries who attended follow-up appointments at the spinal cord injury department of the rehabilitation center. This center is one of the largest rehabilitation facilities, with a 500-bed capacity, providing clinical, educational, and supportive services [17]. The follow-up is scheduled once in one to three months depending on the patient’s condition. The rehabilitation consultant conducts an extensive evaluation of the patient’s medical status, physical examination, functional capabilities, psycho-social health, neurological conditions, medical complications, and muscle strength, tone, mobility, and activities of daily living. Additionally, psychological factors such as mood, coping mechanisms, and social support systems are also examined. The follow-up visit aims to ensure early identification of rehabilitation needs and the optimization of care plans to facilitate recovery and promote long-term independence.

The study used the Arabic version of the validated World Health Organization Quality of Life-BREF (WHOQOL-BREF) questionnaire, which is commonly used to evaluate the HRQoL among the disabled population [10,18]. The Arabic version of the questionnaire has been validated in the Arab population, achieving a Cronbach’s alpha of 0.7 for all domains, suggesting high internal consistency and validity [19]. Inclusion criteria were Saudi males and females with traumatic spinal cord injuries, aged 18 to 50 years, who completed a comprehensive inpatient rehabilitation program and could read and respond to the questionnaire. Exclusion criteria included patients with co-existing brain injuries and those who did not consent to participate.

### 2.2. Sample Size and Data Collection

Data collection was conducted by two trained research personnel who approached the outpatient clinic’s patient registration counter to collect information on visiting SCI patients daily. A non-probability consecutive sampling method was employed to recruit all eligible patients within the designated time frame and study period of two months. The sampling process commenced at the beginning of September 2024 and concluded at the end of October, encompassing a two-month timeframe for data collection. During this period, a total of 75 SCI patients attended the outpatient clinic for a routine follow-up visit at the rehabilitation center. Among these, fifty patients met the inclusion criteria and were invited to participate in the study. With a response rate of 100%, all fifty individuals consented to participate in the study.

Participants were thoroughly informed about the study objectives, and informed consent was obtained prior to the start of data collection. Furthermore, participants were informed of voluntary participation with the option to withdraw at any time without having to face any consequences. Total confidentiality and anonymity were assured, as the only identifier used in the questionnaire was the medical record number obtained from the electronic health records. The questionnaire was self-administered by the patients. The entire process, from obtaining consent to finishing the questionnaire, took about twenty to thirty minutes to complete.

### 2.3. Study Variables and Description of Study Tools

The case report form (CRF) used to collect the data from the health information system (HIS) included the patients’ demographic information, level of injury, and American Spinal Injury Association (ASIA) scale, the Functional Independence Measure (FIM) score, and the number of admissions to inpatient rehabilitation. The dependent variables included WHOQOL-BREF: overall HRQoL, including physical, psychological, social, and environmental components. The independent variables included all the sociodemographic characteristics, level of injury, ASIA scores, FIM scores, and complications.

The WHOQOL-BREF is a standard and validated tool that assesses the HRQoL of SCI patients [10]. This instrument is a condensed version of the original, featuring 24 items across four quality-of-life domains: physical health (7 items), psychological well-being (6 items), social relationships (3 items), and environment (8 items). It utilizes a five-point Likert scale. Scores for each domain were recoded to align with specific interpretations, such as (1 = not at all, 2 = a little, 3 = moderately, 4 = mostly/very much, 5 = completely/extremely). Other scores were classified as follows: (1 = very dissatisfied, 2 = dissatisfied, 3 = neither satisfied nor dissatisfied, 4 = satisfied, 5 = very satisfied). The average score of the items in each domain was used to calculate the overall domain score. Additionally, two items were analyzed independently: the first assessed the individual’s overall perception of health-related quality of life (“How would you rate your quality of life?”) with scores (1 = very poor, 2 = poor, 3 = neither good nor poor, 4 = good, 5 = very good). The second item evaluated the individual’s general perception of their health (“How satisfied are you with your health?”), scoring (1 = very dissatisfied, 2 = dissatisfied, 3 = neither satisfied nor dissatisfied, 4 = satisfied, 5 = very satisfied). In total, the instrument includes 26 items, with items Q3, Q4, and Q26 being reverse-scored [10].

The ASIA scale categorizes spinal cord injuries from A to E, based on motor and sensory assessments. Specifically, ASIA A denotes a complete spinal cord injury with no sensory or motor function preserved. ASIA B indicates no motor function, but some sensory functions may remain below the level of injury. ASIA C includes both sensory and motor functions that are partially preserved, and ASIA D is characterized by the preservation of motor function below the injury level despite being an incomplete injury [20]. Functional evaluation is defined as “The assessment of an individual’s ability to perform self-care and fulfill basic activities of daily living (ADLs)”. The Functional Independence Measure (FIM) was developed in the 1980s to quantify functional capacity and independence [21]. This scale measures the level of difficulty or limitation of each individual. The assessment consists of 13 motor items and five social–cognitive items, evaluating self-care, sphincter management, transfers, locomotion, communication, social interaction, and cognition. It employs a 7-level scale, ranging from total dependence (1) to complete independence (7). The intermediate levels are defined as follows: 6 for modified independence, 5 for supervision or setup, 4 for minimal contact assistance (where the individual performs at least 75% of the effort), 3 for moderate assistance (where the individual performs 50–75% of the effort), and 2 for maximal assistance (where the individual performs 25–50% of the effort).

FIM was further divided into two parts: self-care and mobility. Self-care included eating, grooming, bathing, upper and lower body dressing, and using the toilet, while mobility included bed to wheelchair transfer, toilet transfer, tub–shower transfer, walking, wheelchair mobility, and stairs use [22].

### 2.4. Statistical Analysis

The collected data were transferred to MS Excel, double-checked for accuracy, refined, coded, and prepared for analysis. The scores for the WHOQOL-BREF domains were managed in accordance with WHO guidelines. Each question was coded on a scale of 1 to 5. Questions 3, 4, and 26, being negatively framed, were reverse-coded. Domain scores were then converted to a 4–20 scale by multiplying the mean scores by 4. Subsequently, these scores were transformed to a 0–100 scale using the formula: Transformed Score = (score − 4) × (100/16), interpreted as higher scores mean higher perception. The results were appropriately tabulated. Means and standard deviations were reported for continuous variables, while frequencies and percentages were used for categorical variables. The Shapiro–Wilk normality test revealed that three domains followed a normal distribution, whereas the general questions and the social domain exhibited a non-normal distribution. Based on these findings, differences in study variables related to the three WHOQOL-BREF domains were analyzed using one-way analysis of variance (ANOVA). For the general questions and the social relationships domain that exhibited non-normal distribution, differences were tested using the Mann–Whitney U test or the Kruskal–Wallis test, as appropriate. Correlations were assessed using Spearman’s correlation coefficient. All analyses were performed using SPSS version 25.0 for Windows (IBM Corp., Armonk, NY, USA), with a *p*-value of less than 0.05 considered statistically significant.

## 3. Results

A total of 50 patients, with a mean age of 35 ± 10.46 years, were included in this study. The majority were male (92.0%), 68.0% were unmarried, 44.0% were employed, and 52.0% had attained higher education. Additional details are provided in Table 1.

The spinal cord injury details are shown in Table 2. Most SCIs resulted from motor vehicle accidents (84%), with 54% classified as grade A injuries, and 74% were at the paraplegia level. The average time since SCI was 6.3 ± 6.0 years, ranging from 1 to 35 years. The mean number of admissions was 3.2 ± 1.6, and the average time between SCI and the start of the first rehabilitation program was 16.5 ± 13.2 weeks, ranging from 3 to 56 weeks. The self-care FIM score (32.0 ± 9.3) was higher than the mobility FIM score (25.1 ± 8.4). Regarding the WHOQOL-BREF assessment, the overall HRQoL score was 75.5 ± 24.5, and the general health score was 66.5 ± 31.0. Among the WHOQOL-BREF domains, the psychological domain had the highest score (71.7 ± 17.5), while the physical health domain had the lowest (55.9 ± 18.8). More details are presented in Table 2.

The subjective complications of SCI, as documented in the HIS system, are summarized in Table 3. The most common complications were neurogenic bladder and neurogenic bowel (*n* = 48 each), followed by spasticity (*n* = 31) and neuropathic pain (*n* = 26). Other complications were reported less frequently.

The association between WHOQOL-BREF scores with demographic features is shown in Table 4. No significant differences were found between WHOQOL-BREF scores across all domains and patient characteristics. Although not significant, patients with quadriplegia showed lower scores in most domains. Further details are presented in Table 4.

The associations between WHOQOL-BREF scores and complications associated with spinal cord injury (SCI) are presented in Table 5. The presence of neuropathic pain was significantly associated with lower overall quality of life scores (68.3 ± 26.0 vs. 83.3 ± 20.4; *p* = 0.03), as well as diminished scores in both physical health (50.1 ± 20.8 vs. 62.1 ± 14.3; *p* = 0.02) and psychological health (67.0 ± 17.7 vs. 76.9 ± 16.2; *p* = 0.04). Additionally, patients exhibiting anxiety reported significantly lower overall quality of life scores (53.6 ± 30.4 vs. 79.1 ± 21.8; *p* = 0.02). A significant correlation was also identified between environmental health and the presence of spasticity (59.1 ± 15.8 vs. 69.1 ± 17.5; *p* = 0.04). Furthermore, the existence of depression was correlated with reduced physical health scores. Further details can be found in Table 5.

Table 6 presents significant positive correlations between physical health and the time since injury (*p* = 0.03) as well as self-care FIM scores (*p* = 0.03). No significant correlations were found between age, number of admissions, time between injury and the first rehabilitation program, or mobility FIM scores with the WHOQOL-BREF domains.

## 4. Discussion

### 4.1. Summary Findings

The findings of this study contribute to a deeper understanding of the complex interaction between various factors that influence the HRQoL of SCI patients in Saudi Arabia. Despite significant medical advances and rehabilitation techniques, SCI patients continue to face substantial challenges that affect their overall quality of life. This research emphasizes the multifaceted nature of these challenges and highlights the critical role of comprehensive rehabilitation programs. To summarize, the results showed that patients with SCI have lower HRQOL scores. Anxiety and neuropathic pain were found to be significantly associated with lower overall quality of life among people with spinal cord injury. The lower scores in the physical health domain reflect the severe physical limitations caused by SCI, consistent with previous studies documenting the direct impact of physical disability on HRQoL. However, the psychological domain scored relatively higher, suggesting that mental resilience and coping strategies may develop as patients adapt over time.

### 4.2. Four Domain Scores in the Global Context

The current study reveals that the scores obtained from the WHOQOL-BREF assessment were higher compared to those reported in existing global literature across all domains. For example, a study conducted in India reported mean WHOQOL-BREF scores of 54.05, 58.14, 59.14, and 56.29 for individuals with SCI across the physical, psychological, social, and environmental health domains, respectively [23]. In contrast, a Turkish study indicated an overall average WHOQOL-BREF score of 48.2 ± 12.6, in contrast to the present study’s average score of 75.5 ± 24.5 [24]. Furthermore, a research study in China documented lower mean scores in the WHOQOL-BREF, specifically 46.8, 56.0, 52.7, and 52.4 for the physical, psychological, social, and environmental domains, respectively [25].

These findings suggest that individuals in Saudi Arabia may experience enhanced living standards and increased satisfaction, potentially attributed to the quality of care and support systems in place. Such elevated living standards can be linked to comprehensive government initiatives that include free healthcare services, advanced hospital facilities, well-equipped tertiary care centers, high-quality specialized care—including home visitations—and effective social support networks. In addition, extensive social welfare programs aimed at ensuring financial and social security, coupled with strong community engagement and family-oriented cultural practices, positively influence the overall quality of life and living standards in the region.

### 4.3. Overall Quality of Life

The overall mean HRQoL score in our study was 75.5 ± 24.5. In comparison, a recent study conducted in South Africa reported an overall mean HRQoL score of 62.3 ± 17.9 among 122 individuals with SCI [26]. In our study, complications associated with SCI, particularly the presence of neuropathic pain and anxiety, were found to impair overall HRQoL significantly. The interrelated and vicious loop between pain and anxiety is well documented, with chronic pain adversely affecting functionality, sleep quality, and social engagement, thereby exacerbating anxiety levels [27]. Chronic neuropathic pain encompasses a broad spectrum of life aspects, substantially limiting social, emotional, and physical activities, which in turn is directly correlated with diminished HRQoL. These results align with findings from other research indicating lower HRQoL in patients suffering from chronic pain [28,29]. Interestingly, none of the socio-demographic variables examined revealed a statistically significant association with overall HRQoL scores; however, a trend was noted where female participants and those with lower income levels tend to report lower HRQoL scores.

### 4.4. SCI Complications and HRQoL Scores

As discussed above, the single most important complication that affected most of the domains, including physical and psychological health, was neuropathic pain. Additionally, anxiety and depression also notably affect the psychological well-being of SCI patients. The lower scores in the environmental health domain were found to be linked to spasticity. Several studies support our findings. Prior research indicates that pain, spasticity, and functional dysfunction negatively correlate with quality of life and satisfaction, while also increasing psychological distress [30,31].

Interestingly, the duration since spinal cord injury has been linked to improved physical quality of life. This may be due to the development of coping mechanisms and adaptive strategies over time. However, previous literature provides conflicting reports about the relationship between quality of life and time. Cardile and colleagues noted an improvement in quality of life among spinal cord injury patients over time, attributing this to factors such as optimism, coherence, and improved psychological dynamics [28]. In contrast, Chang observed a decline in physical quality of life [24]. Nonetheless, caution should be exercised when interpreting these findings, as various factors—including age, injury severity, comorbidities, and other complications—may influence the results. Social relationships and environmental factors also play crucial roles. Although not statistically significant, the trends suggest that individuals with better social support and more accessible environments tend to report higher HRQoL. This finding is consistent with the literature suggesting that social integration and support systems are pivotal in enhancing the HRQoL for individuals with chronic disabilities [28].

### 4.5. Policy and Practice Implications

SCI patients’ rehabilitation necessitates a reevaluation of the existing care model by policymakers, with an emphasis on comprehensive pain management that incorporates a multidisciplinary approach. The implications for clinical practice and health policy highlight the importance of evolving an integrated, long-term care strategy that prioritizes self-efficacy and psychosocial support, ultimately aiming to enhance overall quality of life for patients.

Utilizing patient-reported outcome measures during routine follow-up visits can facilitate a thorough assessment of patient needs, enable effective monitoring of outcomes, and allow for the provision of tailored interventions. Furthermore, enhancing public awareness and promoting a disability-friendly society can significantly benefit individuals with SCI. Finally, policymakers must support ongoing research initiatives focused on improving the health-related quality of life for patients with spinal cord injuries.

## 5. Strengths and Limitations

This study has several strengths. In the absence of regional data on the HRQoL in SCI patients, the present study addresses the knowledge gaps in areas like the health-related quality of life of spinal cord injury patients and the key determinants associated with poor scores. The data was collected from the department specializing in spinal cord injury at a tertiary care rehabilitation center, where patients from all over the country visit the center.

Some limitations should be considered when interpreting the findings. First, the relatively small sample size (50 participants) limits the generalizability of the results to the broader population of SCI patients in Saudi Arabia. Second, the study was conducted in a single tertiary care center, which may not reflect the experiences of SCI patients receiving care in other facilities or regions. Third, the cross-sectional design captures HRQoL at a single point in time, making it difficult to assess changes over time or the impact of rehabilitation interventions longitudinally, and its inability to infer causality. Additionally, the study relied on self-reported data, which may be influenced by recall bias or social desirability. The consecutive method of sampling may have introduced selection bias due to the absence of randomization. The study did not take measures to handle confounders. Finally, while the WHOQOL-BREF is a validated tool, it may not capture all culturally specific or nuanced factors affecting HRQoL in the Saudi context, although the Arabic version has been validated in Arab population.

## 6. Conclusions

Neuropathic pain, anxiety, and depression, along with spasticity, adversely affect the health-related quality of life of individuals with SCI. This research emphasizes a comprehensive but tailored approach in the rehabilitation of SCI patients, considering not only physical health but also the psychological, social, and environmental dimensions involved in recovery. Customized interventions that cater to the unique needs and challenges faced by SCI patients can greatly improve their rehabilitation outcomes and overall quality of life. Future longitudinal studies involving a larger sample size and across multiple health centers are highly recommended, with a focus on key determinants, pain management methods and coping strategies, psychosocial research, and the impact of interventions.

## Figures and Tables

**Table 1:** Characteristics of the study sample.

	Frequency	Percent
Gender		
Male	46	92.0
Female	4	8.0
Marital status		
Unmarried	34	68.0
Married	16	32.0
Place of residency		
Western	8	16.0
Southern	10	20.0
Northern	6	12.0
Eastern	10	20.0
Central	16	32.0
Source of income		
Working	22	44.0
Supported by family	9	18.0
Social Affairs	5	10.0
Retired	14	28.0
Education level		
Primary level	24	48.0
Higher level	26	52.0

**Table 2:** Descriptive statistics of the study variables related to SCI, FIM score, and WHOQOL-BREF domains.

	Frequency	Percent
Cause of SCI		
Accident	42	84.0
Fall	8	16.0
Grade of SCI		
Grade A	27	54.0
Grade B	8	16.0
Grade C	7	14.0
Grade D	8	16.0
Level of SCI		
Paraplegia	37	74.0
Quadriplegia	13	26.0
	Mean ± SD	
Time since injury (years)	6.3 ± 6.0	
Number of admissions	3.2 ± 1.6	
Time between injury and 1st rehabilitation program (weeks)	16.5 ± 13.2	
Self-care FIM	32.0 ± 9.3	
Mobility FIM	25.1 ± 8.4	
Overall quality of life (HRQoL)	75.5 ± 24.5	
Overall general health (GH)	66.5 ± 31.0	
Physical health (Domain 1)	55.9 ± 18.8	
Psychological (Domain 2)	71.7 ± 17.5	
Social relationships (Domain 3)	66.2 ± 22.6	
Environment (Domain 4)	62.9 ± 17.0	

**Table 3:** Complications of the SCI as reported by patients (multiple choice).

	Present	Not Present
Spasticity	31 (62.0)	19 (38.0)
Neurogenic bladder	48 (96.0)	2 (4.0)
Heterotopic ossification	6 (12.0)	44 (88.0)
Depression	10 (20.0)	40 (80.0)
Neurogenic bowel	48 (96.0)	2 (4.0)
Osteoporosis	18 (36.0)	32 (64.0)
Pressure ulcer	16 (32.0)	34 (68.0)
Anxiety	7 (14.0)	43 (86.0)
Neuropathic pain	26 (52.0)	24 (48.0)
Respiratory complication	1 (2.0)	49 (98.0)
Autonomic dysreflexia	12 (24.0)	38 (76.0)

**Table 4:** Associations between WHOQOL-BREF with the demographic characteristics and cause, level, and grade of the SCI injury.

		Overall Quality of Life		Overall General Health		Physical Health		Psychological Health		Social Relationships		Environment Health	
		Mean ± SD	*p*	Mean ± SD	*p*	Mean ± SD	*p*	Mean ± SD	*p*	Mean ± SD	*p*	Mean ± SD	*p*
Gender	Male	76.1 ± 24.7	0.50 *	66.3 ± 31.7	0.95 *	55.7 ± 18.2	0.88	72.5 ± 16.8	0.33	65.4 ± 23.2	0.45 *	63.2 ± 17.2	0.67
	Female	68.8 ± 23.9		68.8 ± 23.9		57.1 ± 28.7		63.5 ± 26.0		75.0 ± 11.8		59.4 ± 15.1	
Marital status	Unmarried	75.7 ± 22.6	0.86 ^a^	66.2 ± 28.8	0.68 ^a^	58.6 ± 18.0	0.13	71.9 ± 19.4	0.91	68.1 ± 17.6	0.58 ^a^	63.0 ± 17.1	0.96
	Married	75.0 ± 28.9		67.2 ± 36.2		50.0 ± 19.7		71.4 ± 13.3		62.0 ± 31.0		62.7 ± 17.2	
Education level	Primary level	79.2 ± 24.1	0.25 ^a^	61.5 ± 33.0	0.29 ^a^	51.2 ± 16.1	0.18	71.7 ± 16.8	0.98	63.5 ± 21.1	0.43 ^a^	62.4 ± 16.1	0.84
	Higher level	72.1 ± 24.8		71.2 ± 28.9		60.2 ± 20.4		71.8 ± 18.5		68.6 ± 24.1		63.3 ± 18.1	
Source of income	Working	78.4 ± 19.4	0.70 ^a^	68.2 ± 31.0	0.38 ^a^	60.6 ± 19.3	0.37	74.1 ± 18.9	0.12	71.6 ± 20.7	0.11 ^a^	65.8 ± 20.4	0.38
	Supported by family	75.0 ± 21.7		80.6 ± 16.7		56.3 ± 16.1		72.2 ± 15.0		70.4 ± 17.7		61.1 ± 15.6	
	Social Affairs	60.0 ± 37.9		50.0 ± 35.4		48.6 ± 16.5		54.2 ± 21.2		55.0 ± 26.1		51.3 ± 12.4	
	Retired	76.8 ± 28.5		60.7 ± 35.0		50.8 ± 20.1		74.1 ± 13.1		58.9 ± 25.8		63.6 ± 12.1	
Cause of SCI	Accident	75.0 ± 25.3	0.88 *	66.1 ± 31.1	0.82 *	57.1 ± 19.7	0.30	72.6 ± 17.8	0.42	66.9 ± 22.1	0.52 *	62.9 ± 17.3	0.94
	Fall	78.1 ± 20.9		68.8 ± 32.0		49.6 ± 12.1		67.2 ± 16.3		62.5 ± 26.4		62.5 ± 16.1	
Level of SCI	Paraplegia	75.0 ± 24.3	0.76 *	67.6 ± 30.5	0.70 *	58.6 ± 18.7	0.08	73.8 ± 16.9	0.17	68.5 ± 21.3	0.23 *	65.4 ± 17.9	0.07
	Quadriplegia	76.9 ± 25.9		63.5 ± 33.3		48.1 ± 17.4		66.0 ± 18.7		59.6 ± 25.6		55.8 ± 11.8	
Grade of SCI	Grade A	75.9 ± 27.3	0.25 ^a^	63.0 ± 31.3	0.59 ^a^	55.4 ± 19.2	0.46	70.1 ± 20.4	0.15	63.3 ± 22.1	0.05 ^a^	61.6 ± 19.0	0.12
	Grade B	78.1 ± 16.0		75.0 ± 40.1		60.7 ± 22.0		81.2 ± 12.4		82.3 ± 10.4		74.2 ± 10.3	
	Grade C	85.7 ± 19.7		71.4 ± 22.5		61.2 ± 18.4		77.4 ± 7.2		71.4 ± 20.9		64.3 ± 14.1	
	Grade D	62.5 ± 23.1		65.6 ± 29.7		47.8 ± 13.9		63.0 ± 13.3		55.2 ± 28.1		54.7 ± 12.9	

* Mann–Whitney U test was used; ^a^ Kruskal–Wallis test was used; p-value was considered significant at p < 0.05.

**Table 5:** Associations between the reported SCI complications and WHOQOL-BREF.

		Overall Quality of Life β		Overall General Health β		Physical Health		Psychological Health		Environment Health	
		Mean ± SD	*p*	Mean ± SD	*p*	Mean ± SD	*p*	Mean ± SD	*p*	Mean ± SD	*p*
Spasticity	No	71.1 ± 28.0	0.42 ^β^	76.3 ± 29.4	**0.05 ^β^**	60.7 ± 20.9	0.15	71.5 ± 19.2	0.93	69.1 ± 17.5	**0.04**
	Yes	78.2 ± 22.1		60.5 ± 30.8		52.9 ± 17.0		71.9 ± 16.8		59.1 ± 15.8	
Neurogenic Bladder	No	75.0 ± 0.0	0.79	75.0 ± 0.0	0.87	55.4 ± 12.6	0.97	68.8 ± 8.8	0.80	65.6 ± 0.0	0.25
	Yes	75.5 ± 25.0		66.1 ± 31.6		55.9 ± 19.1		71.9 ± 17.9		62.8 ± 17.3	
Heterotopic Ossification	No	76.1 ± 25.3	0.43	65.9 ± 30.5	0.62	56.7 ± 18.6	0.37	72.6 ± 18.0	0.34	62.4 ± 17.8	0.42
	Yes	70.8 ± 18.8		70.8 ± 36.8		49.4 ± 20.4		65.3 ± 13.6		66.2 ± 8.9	
Depression	No	76.9 ± 22.2	0.66	65.0 ± 31.4	0.51	58.5 ± 17.5	0.04	72.6 ± 17.5	0.49	63.1 ± 17.3	0.83
	Yes	70.0 ± 32.9		72.5 ± 29.9		45.4 ± 21.0		68.3 ± 18.3		61.9 ± 16.3	
Neurogenic Bowel	No	75.0 ± 0.0	0.79	75.0 ± 0.0	0.87	55.4 ± 12.6	0.97	68.8 ± 8.8	0.80	65.6 ± 0.0	0.25
	Yes	75.5 ± 25.0		66.1 ± 31.6		55.9 ± 19.1		71.9 ± 17.9		62.8 ± 17.3	
Osteoporosis	No	75.0 ± 24.6	0.79	67.2 ± 31.4	0.82	55.2 ± 19.3	0.76	72.4 ± 16.3	0.73	65.6 ± 16.6	0.12
	Yes	76.4 ± 25.0		65.3 ± 31.1		56.9 ± 18.4		70.6 ± 20.0		58.0 ± 17.0	
Pressure Ulcer	No	74.3 ± 25.0	0.60	68.4 ± 29.7	0.61	56.0 ± 18.2	0.94	73.0 ± 16.8	0.45	64.8 ± 17.8	0.24
	Yes	78.1 ± 23.9		62.5 ± 34.2		55.6 ± 20.6		69.0 ± 19.4		58.8 ± 14.8	
Anxiety	No	79.1 ± 21.8	**0.02**	68.0 ± 29.5	0.49	57.5 ± 17.9	0.13	72.4 ± 18.2	0.53	63.2 ± 17.0	0.77
	Yes	53.6 ± 30.4		57.1 ± 40.1		45.9 ± 22.6		67.9 ± 12.9		61.2 ± 17.9	
Neuropathic Pain	No	83.3 ± 20.4	**0.03**	74.0 ± 25.0	0.16	62.1 ± 14.3	**0.02**	76.9 ± 16.2	**0.04**	67.6 ± 16.1	**0.05**
	Yes	68.3 ± 26.0		59.6 ± 34.7		50.1 ± 20.8		67.0 ± 17.7		58.5 ± 16.9	
Autonomic Dysreflexia	No	73.0 ± 25.6	0.23 ^β^	67.8 ± 29.0	0.82	56.3 ± 18.7	0.77	72.1 ± 17.4	0.77	63.9 ± 17.9	0.45
	Yes	83.3 ± 19.5		62.5 ± 37.7		54.5 ± 19.9		70.5 ± 18.8		59.6 ± 13.8	

*Note: Since the social relationships domain did not show any significant results, the domain was removed from the table. ^β^ denotes Mann-Whitney U test was used. p-value was considered significant at p < 0.05.*

**Table 6:** Correlations between age, time of injury, number of admissions, FIM scores, with the WHOQOL-BREF.

		Overall Quality of Life	Overall General Health	Physical Health	Psychological Health	Social Relationships	Environment Health
Age (years)	Correlation	0.176	0.040	−0.195	0.065	−0.235	−0.179
	*p*-value	0.22	0.78	0.17	0.65	0.10	0.21
Time since injury	Correlation	0.076	0.131	0.292 *	0.203	0.117	0.021
	*p*-value	0.60	0.36	**0.03**	0.15	0.41	0.88
Number of admissions	Correlation	0.114	0.242	0.230	0.064	−0.013	−0.015
	*p*-value	0.43	0.09	0.10	0.66	0.92	0.91
Time between injury and 1st rehab. program	Correlation	−0.123	0.011	0.035	0.130	−0.238	−0.020
	*p*-value	0.39	0.93	0.81	0.36	0.09	0.88
Self-care FIM	Correlation	−0.120	0.188	0.306 *	0.142	0.270	0.214
	*p*-value	0.40	0.19	**0.03**	0.32	**0.05**	0.13
Mobility FIM	Correlation	−0.203	0.063	0.146	−0.015	0.125	0.108
	*p*-value	0.15	0.66	0.31	0.92	0.38	0.45

Spearman Correlation Coefficient test was used; * p-value was considered significant at p < 0.05.

## Data Availability

The data presented in this study are available on request from the corresponding author since the data are not publicly available due to privacy or ethical restrictions.
